# Role of Chemerin/ChemR23 axis as an emerging therapeutic perspective on obesity-related vascular dysfunction

**DOI:** 10.1186/s12967-021-03220-7

**Published:** 2022-03-22

**Authors:** Yingying Xie, Ling Liu

**Affiliations:** 1 Department of Cardiovascular Medicine, The Second Xiangya Hospital, Central South University, Changsha, China; 2 Research Institute of Blood Lipid and Atherosclerosis, Central South University, Changsha, China; 3 Modern Cardiovascular Disease Clinical Technology Research Center of Hunan Province, Changsha, China; 4 Cardiovascular Disease Research Center of Hunan Province, Changsha, China

**Keywords:** Chemerin, ChemR23, White adipose tissue, Adipokine, Obesity, Vascular dysfunction

## Abstract

Sufficient epidemiological investigations demonstrate that there is a close correlation between obesity and vascular dysfunction. Nevertheless, specific mechanisms underlying this link remain currently unclear. Given the crucial and decisive role of vascular dysfunction in multitudinous diseases, various hypotheses had been proposed and numerous experiments were being carried out. One recognized view is that increased adipokine secretion following the expanded mass of white adipose tissue due to obesity contributes to the regulation of vascular function. Chemerin, as a neo-adipokine, whose systemic level is elevated in obesity, is believed as a regulator of adipogenesis, inflammation, and vascular dysfunction via binding its cell surface receptor, chemR23. Hence, this review aims to focus on the up-to-date proof on chemerin/chemR23 axis-relevant signaling pathways, emphasize the multifarious impacts of chemerin/chemR23 axis on vascular function regulation, raise certain unsettled questions to inspire further investigations, and explore the therapeutic possibilities targeting chemerin/chemR23.

## Introduction

Over the years, obesity has become a worldwide public health incident [1, 2], which is mainly caused by the accumulation of white adipose tissue (WAT) [3]. Besides, obesity is also an acknowledged predisposing and risk factor for types of disorders, including diabetes, dyslipidemia, vascular dysfunction and so on [4]. Vascular dysfunction then leads to multi-system diseases, such as cardiovascular system diseases (atherosclerosis, hypertension) [5, 6], respiratory system diseases (pulmonary hypertension, adult respiratory distress syndrome) [7, 8], digestive system diseases (Budd-Chiari syndrome, severe acute pancreatitis) [9, 10] and so on.

WAT is a well-acknowledged human endocrine organ with the secretion of various adipokines (i.e. adipocytokines), in addition to serving as energy storage [11]. Adipokines are the conditioning agents of adipogenesis, vascular function, glucose and insulin metabolisms [12–15], whose regulating ability becomes more pronounced with the expanded mass of WAT due to obesity. Chemerin is also a protein whose systemic level will increase in obesity and plays an extremely important role in regulating vascular function through binding its receptor, chemR23. Notably, chemerin can be activated in obesity, transforming from inert prochemerin to activated chemerin [16].

Interestingly, chemerin is more highly expressed in perivascular adipose tissue (PVAT) than in subcutaneous or visceral adipose tissue [17], which provides a mechanistic explanation for the regulation of vascular function by chemerin, partly due to the natural location advantage and the absence of mechanical barrier between PVAT and blood vessels [18]. Evidence had shown that chemerin/chemR23 axis exerted significant effects on the regulation of vascular function, but the detailed mechanisms and pathways mediated by this axis remained controversial.

In this review, we introduce the formation of chemerin via COOH-terminal processing and the alteration of expressions of both chemerin and chemR23 in obesity. Furthermore, the role of chemerin/chemR23 axis in vascular dysfunction, especially when the body is in the state of obesity is described in detail.

## Chemerin feature and processing

Chemerin is a 16 KDa protein and encoded by tazarotene induced gene 2 (i.e. retinoic acid receptor responder 2). It was initially reported as a synthetic retinoid gene in psoriatic skin lesions in 1997 [19, 20]. In previous studies, chemerin was identified as a natural ligand of orphan guanosine 5'-triphosphate (GTP)-binding protein-coupled receptors (GPCR), also known as chemokine like receptor 1 (CMKLR1) or chemR23 [18]. There exist three phases before chemerin evolves into active modalities: pre-prochemerin, prochemerin, and chemerin stages [22]. So-called pre-prochemerin (1–163) is a 163-amino-acid long protein without biological activity and directly encoded by tazarotene induced gene 2. It transforms into prochemerin (21–163), a 143-amino-acid long protein with low biological activity, by getting rid of 20 NH_2_-terminal amino acids in a proteolytic cleavage way [20, 23]. And prochemerin undergoes extracellular COOH-terminal processing by certain proteases [24], ending up as chemerin with higher biological activity and chemR23 receptor binding ability [20].

It is worth mentioning that different types of proteases simultaneously cut prochemerin into diverse length products that differ in COOH-terminal amino acids and potency for chemR23 activation. For instance, the first enzymes shown to activate prochemerin are called elastase and cathepsin G [24], both belonging to neutrophil proteases species. The former removes the 6, 8, or 11 COOH-terminal amino acids to produce three forms, chemerin (21–157), (21–155), or (21–152), respectively [21]. Among them, chemerin (21–157) holds the highest activity. While the latter clears the 7 COOH-terminal amino acids to produce one fragment, chemerin (21–156) [25], which is the second most active form next to chemerin (21–157), and both are the two active forms in the human body.

Prochemerin is also cut into chemerin (21–158) with low activity mediated by tryptase and plasmin through sweeping the 5 COOH-terminal amino acids away. Chemerin (21–158) undergoes the second processing to complement this activity by carboxypeptidases N or B (CPN or CPB), ultimately forming chemerin (21–157) [26, 27]. This illustrates that the segments formed by the COOH-terminal processing might be also the substrates in the subsequent processing.

In addition to the above typical proteases leading to the formation of various fragments, there are some enzymes (e.g., mast cell chymase and angiotensin-converting enzyme) having been reported to cleave certain fragments to generate relatively inactive modalities, including chemerin (21–154) and chemerin (21–155) [28, 29].

Taken together, chemerin is conscripted to undergo proteolytic cleavages to remove amino acids located in NH_2_-terminal and COOH-terminal by multitudinous proteases before the formation of diverse isoforms (Table 1), which differ in their activities. Most of them hold a low activity or no activity, even antagonizing the active isoforms, generally referring to chemerin (21–157). This manifests that a complex regulatory network controls chemerin bioactivity.

Noticeably, excessive chemerin tends to be activated when the body is in the state of obesity with the accelerated COOH-terminal processing. To some extent, it is a hint that chemerin seems to have inextricable relation to obesity.

## Chemerin receptor types and characteristics

**Fig. 1:**
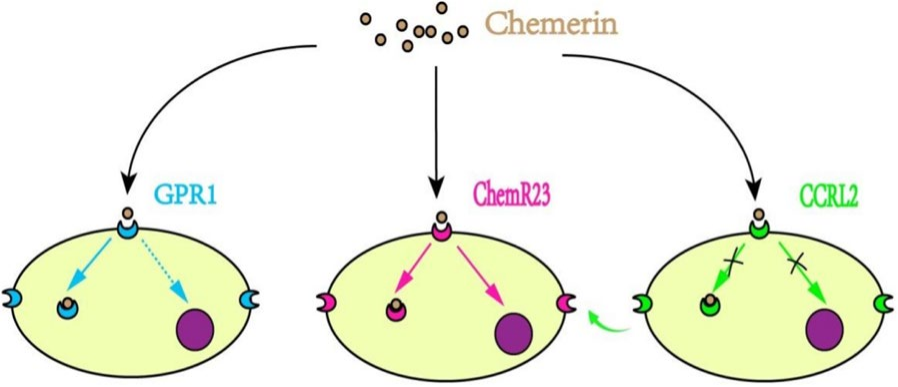
Distinctions of the three receptors for chemerin. ChemR23, GPR1 and CCRL2 are cell-surface receptors, and all of them have a high affinity for chemerin. ChemR23 leads to strong signaling and internalization of the chemerin–receptor complex. GPR1 leads to weak signaling but also displays equal internalization as chemR23. CCRL2 does not signal nor internalizes but might pass on chemerin to functional chemR23 of nearby cells

The chemerin receptors that have been recognized and understood mainly include chemR23(i.e. GPCR or CMKLR1), G protein-coupled receptor (GPR)1 and chemokine (C–C motif) receptor-like (CCRL)2. They are all located at the surface of cells but distinct from each other in the affinity of binding to chemerin, signaling, and internalization of the chemerin-receptor complex (Fig. 1).

### ChemR23

Chemerin is a natural ligand of chemR23. As the well-deserved nature receptor with the highest affinity binding, efficient signaling and internalization, chemR23 is presently the only one reflecting the veritable activity of chemerin and accurately representing chemerin targeting sites among the three receptors. Chemerin active forms were observed only through measuring the activity on chemR23-expressing cells [25]. In other words, where there is chemR23, there is chemerin combining target. Besides, chemR23 is structurally relevant to a suite of chemokines, such as complement fragments (e.g., C5a, C3a) and prostaglandin D2 [30] which also cover GPR1 and the orphan receptors GPR32 and GPR33 [25].

Furthermore, chemR23 has a second ligand named resolvin E1 (RvE1), a new bioactive oxygenated product of the essential fatty eicosapentaenoic acid (EPA), which is one of the main types of omega-3 polyunsaturated fatty acids (ω-3 PUFAs), existing in fish oils [31, 32]. Based on the powerfully anti-inflammatory role of ω-3 PUFAs in cardiovascular diseases, the combination of chemR23 and RvE1 is increasingly taken for a salutary one, differing from chemerin/chemR23 axis, even quite the opposite [33, 34].

### GPR1 and CCRL2

In addition to chemR23, GPR1 and CCRL2 are universally acknowledged as two other receptor types of chemerin [35]. GPR1 structurally resembles ChemR23, which is concretely embodied in a 37% similar sequence identity between them [36]. That is why GPR1 is taken as a potential candidate in binding and activating chemerin in addition to chemR23. Meanwhile, GPR1 was mapped genome-wide of human loci for essential hypertension, involving the British Genetics of Hypertension (BRIGHT) study in 2003 [37]. Regrettably, despite high-affinity binding, GPR1 shows weak signaling. It has been found that chemerin elicits potent constrictor actions via chemR23, not GPR1 [38].

CCRL2 is also referred to as Eo1 in mice, and chemokine receptor (HCR) in humans. Though previous thought of as leukocyte chemoattractant receptor binding the chemokines C–C motif chemokine ligand (CCL)2, CCL5, CCL7, and CCL8 [39], CCRL2 is latterly depicted as a third receptor for chemerin with high-affinity binding [40]. Unlike the first two receptors, CCRL2 shows no signaling or internalization. Whereas, the conclusion cannot be drawn that CCRL2 is disqualified as the receptor to bind chemerin and activate chemerin/chemR23 axis. Instead, CCRL2 is proposed for elevating the local concentration of chemerin and presenting chemerin to GPR1 and chemR23 nearby, implying its role as a regulator of chemerin concentration and a mediator of chemerin transfer [40].

It appears that the other two receptors, GPR1 and CCRL2 are likely to participate in the peculiarity manifestations of chemerin, but both take effect with mechanisms relying on chemR23 more or less.

## The changes of chemerin/chemR23 axis in obesity

### Increased expression of chemerin in obesity

#### Evidence from clinical observations

##### Chemerin concentrations in obese patients

Chemerin is a secreted protein that is detected in the plasma or serum. Generally speaking, it is a trend that women and the elderly have higher circulating chemerin concentrations than men and the young [41, 42]. Though chemerin concentration partly varies with age and gender, it always fluctuates from 90 to 200 ng/mL in non-obese populations. Owing to the varying degree of obesity, the range of chemerin concentration fluctuation is too large to be represented simply by a numerical value in obese populations.

Circulating chemerin concentration is certainly higher in obese patients than lean ones, which has been confirmed by numerous studies [43, 44]. Obesity is often classified into different grades by body mass index (BMI), so, previous studies elucidated that plasma chemerin concentration positively correlates with BMI [43]. A rising view is that BMI puts more emphasis on the amount of subcutaneous adipose tissue (SAT), not visceral adipose tissue (VAT), which was confirmed by stepwise multiple regression analysis [45]. And VAT is better represented by waistline or waist-to-hip ratio (WHR). Given that chemerin was reported to be expressed more in VAT, particularly in PVAT, than in SAT [46–48], relevant evidence supported serum chemerin concentration was also positively correlated with waistline or WHR, both of which were more representative of circulating chemerin than BMI [42, 49]. Besides, studies about aerobic exercise [50] and losing weight [51] as lateral proofs verified that chemerin can be reduced through the cut-down of WAT mass.

##### Chemerin mRNA and protein expressions in obese patients

Similar to the up-regulation of circulating chemerin concentration due to obesity, chemerin protein expression is increased in tissues from obese patients. A study described that compared with the WAT of non-obese individuals, the WAT from obese donors produced higher chemerin protein mass [52]. In addition to WAT, other tissues like skeletal muscle also show a similar trend [52].

The mRNA expression of chemerin is also elevated in obese individuals. It was observed that chemerin mRNA expression was dramatically higher expressed in omental adipose tissue and SAT of obese patients, and weight loss induced by bariatric surgery led to a lower mRNA expression of chemerin than before [43]. Additionally, chemerin mRNA expression was elevated significantly in obese patients with non-alcoholic fatty liver disease [53]. In 56 morbidly obese women (BMI > 40 kg/m^2^), hepatic chemerin mRNA expression was induced [53, 54]. Increased expressions of other inflammatory factors, such as tumor necrosis factor α (TNFα) and lipopolysaccharide, were also detected in WAT of obese patients when chemerin expression was elevated. It indicates that inflammation could enhance chemerin production in WAT and other tissues [49, 55].

The conjecture that circulating chemerin protein depends on chemerin mRNA expression in WAT is reasonable in many cases. However, chemerin mRNA and protein expressions are not always synchronously modulated. For instance, chemerin mRNA expressions in SAT and VAT seemed not to be associated with serum chemerin concentration [56]. A study even revealed the negative correlation of chemerin mRNA expression in SAT with the systemic level of chemerin [57]. These seemingly contradictory experimental results manifest, to some extent, that chemerin is partly regulated by unknown post-transcriptional mechanisms.

#### Evidence from animal experiments

##### Chemerin concentration in obese animals

There are usually two types of experimental models associated with obesity. The first is mice with genetic deficiency, such as db/db mice (leptin receptor-deficient) and ob/ob mice (leptin-deficient). The second is obese mice induced by a high-fat diet (HFD), called diet-induced-obesity (DIO) mice. Circulating chemerin concentration in db/db mice was approximately two-fold higher than that in wild-type (WT) mice [58]. Female DIO mice had higher systemic chemerin concentrations compared to mice fed the control chow diet [59]. Moreover, compared with db/db mice treated with vehicle, CCX832 (chemR23 antagonist)treatment decreased body weight of db/db mice, accompanied by reduction of chemR23 protein expression. It is suggested that chemerin/chemR23 axis could be involved in the development of obesity.

##### Chemerin protein and mRNA expressions in obese animals

In terms of tissues, protein expression of chemerin varies with different parts, the highest in WAT and liver [61], followed by brown adipose tissue, lung, heart, ovary, and kidney [61]. Female DIO mice had markedly elevated chemerin protein expression in SAT and VAT [62]. Chemerin protein expression was up-regulated prominently in the gonadal adipose tissue of ob/ob mice [63]. Of interest, the level of chemerin protein expression in WAT of obese animal models was observed to go down after injection of 0.5 µg/g leptin, demonstrating that leptin resistance may be one of the reasons for elevated chemerin protein expression in obesity.

Hepatic chemerin mRNA expression of db/db mice was twofold higher than that of WT mice [58]. Hepatic chemerin mRNA expression was also induced in DIO mice [64]. In ob/ob mice, chemerin mRNA was not significantly changed in the liver, but elevated in the skeletal muscle [58], indicating the participation of chemerin in skeletal muscle insulin resistance via underlying mechanisms.

#### Evidence from cell researches

In physiological conditions, besides mature adipocytes and preadipocytes, chemerin was expressed in types of cell populations, like epithelial cells [65], endothelial cells [66, 67], fibroblasts [68], chondrocytes, and platelets [27], because those cells contain prochemerin transcripts. Sherd of evidence manifested that platelets also stockpiled a small amount of chemerin and released it under the stimulation of platelet-activating factors as platelet activity fluctuated [27].

At the cellular level, obesity is characterized by an increase in adipocyte cell size (hypertrophy), adipocyte cell number (hyperplasia), or both [27]. Studies showed that chemerin was detected on the 6th day (the early stage of differentiation) and increased dramatically on the 9th day (the end of differentiation) in 3T3-L1 preadipocytes, revealing the up-regulated expression of chemerin during adipogenic differentiation.

### Increased expression of chemR23 in obesity

#### Evidence from obese patients

Interestingly, both chemR23 protein and mRNA expression show similar trends to chemerin in obese patients. ChemR23 protein expression was increased prominently in adipocytes and skeletal muscle cells of obese subjects when compared to lean, healthy ones [52]. Hepatic chemR23 mRNA expression was likewise induced markedly in patients suffering from non-alcoholic fatty liver disease [53]. ChemR23 mRNA expression in VATvwas dramatically higher in obese patients than that in lean volunteers. What’s more, there was a significant correlation and synchronism between chemerin and chemR23 mRNA expressions [53]. Bariatric surgery-induced weight loss reduced chemR23 protein and mRNA expressions in obese patients [43]. These findings were obtained from tissue samples of obese individuals. In order to further explore its possible mechanism, related investigation needs to be carried out in obese animal models.

#### Evidence from animal experiments

Through experimental animals, scientists can know more about the tissue distribution of chemR23. Under physiological conditions, chemR23 is expressed in WAT and hematopoietic tissues such as the thymus, bone marrow, spleen, fetal liver, and lymphoid organs [4, 69, 70]. While in obese animals, chemR23 protein expression in WAT was affected a lot. For example, chemR23 protein expression was increased markedly in SAT and VAT of obese rodents, including female DIO mice [59], Psammomys obesus [59], db/db mice and ob/ob mice [63]. What’s more, chemR23 protein expression was even up to five times higher than that of WT mice in epididymal adipose tissue of ob/ob mice. Interestingly, the injection of 0.5 µg/g leptin reduced chemR23 protein expression in WAT of ob/ob and db/db mice [63], indicating that leptin deficiency or resistance partly contributes to the elevated chemR23 protein expression in WAT of obese animals.

#### Evidence from cell researches

ChemR23 is expressed in various types of cells, covering blood monocytes, monocyte-derived human macrophages [65], immature dendritic cells [23, 71], plasmacytoid dendritic cells (pDCs) [72], microglial cells, and natural killer cells [73], and low levels in non-irritant CD4^+^ T lymphocytes, polymorphonuclear cells [73], even leukocyte population, where chemerin is not expressed.

Although no increase in the protein and mRNA expressions of chemr23 was observed in 3T3-L1 preadipocytes [59]. The knock-down even loss of chemR23 in 3T3-L1 preadipocytes indeed obstructed adipogenesis through lowering gene expression of adipogenesis like peroxisome proliferator-activated receptor-γ and sterol regulatory element-binding protein 2 [50, 74, 75]. It demonstrates that chemR23 promoted adipogenesis and further led to fat accumulation through enhancing capacity rather than expressions.

### Increased activity of chemerin in obesity

The biological activity of chemerin is influenced as well as its expression by obesity. As mentioned above, chemerin becomes the most active form, chemerin(21–157), through COOH-terminal processing. By using specific enzyme-linked immunosorbent assays for different chemerin forms, chemerin (21–157) concentration in obese subjects was 1000-fold higher than that in non-obese subjects, accompanied by the increase of plasma C-reactive protein (CRP) level. This indicates that chemerin activity increased greatly in obesity perhaps by inflammation-mediated COOH-terminal processing [16].

## Vascular dysfunction mediated by chemerin/chemR23 axis in obesity

Blood vessels are the largest network structure of the human body, mainly composed of the intima, media, adventitia, and PVAT surrounding the adventitia [52]. Endothelial cells (ECs) from intima and vascular smooth muscle cells (VSMCs) from media are recognized as the most pivotal cell populations for normal vascular function. In addition, PVAT attracts much attention recently because diverse adipokines from PVAT regulate vascular function more quickly than those from adipose tissue in other areas [76]. Thus, ECs, VSMCs, and PVAT maintain vascular homeostasis together. In other words, alters of structures or features of any one of them could induce vascular dysfunction.

### Dysfunction of PVAT

PVAT is a double-edged sword in regulating vascular function. Under physiological conditions, PVAT often serves as mechanical support for blood vessels, with stable adipokines secretions. Immunohistochemistry, quantitative real-time polymerase chain reaction and western blot had validated a robust expression of chemerin in PVAT. But in vascular pathologies caused by detrimental states like obesity, PVAT expands in volume, along with increased secretions of adipokines [77, 78]. For example, chemerin and chemR23 protein expressions were higher in PVAT of DIO rats after a four-week HFD [48]. Chemerin antisense oligonucleotides (ASO) with whole-body activity reduced chemerin in PVAT and partially reversed the chemerin/chemR23 axis-induced vascular dysfunction [79]. Broadly speaking, the currently ample evidence supports that chemerin is such a typical adipokine that elevates in obese PVAT and then contributes to vascular dysfunction by further acting on the constituent cells of blood vessels like ECs and VSMCs via various mechanisms and pathways.

### Endothelial dysfunction

#### Enhanced ECs proliferation and migration capacity

Proliferation and migration of ECs driven by proangiogenic molecules lead to angiogenesis, which is the pathophysiological basis of some diseases, such as metastasis of the tumor, atherosclerosis [80], and so on. A mass of in-vitro experiments had manifested that chemerin/chemR23 axis stimulated capillary-like structure formation via promoting the proliferation of ECs, and chemerin functioned as a chemoattractant for ECs to hasten migration [80, 81]. Chemerin/chemR23 axis-induced angiogenesis depends on p38 mitogen-activated protein kinase (MAPK) and extracellular regulated protein kinases (ERK) 1/2 pathway in human umbilical vein endothelial cells [66, 82, 83]. Matrix metalloproteinases-2/9 were also found to get involved in the proliferation and migration of ECs through degrading extracellular matrix in a chemerin dose-dependent way [66]. Besides, new evidence suggested that chemerin/chemR23 axis promoted angiogenesis through enhanced autophagy. It illustrates that chemerin/chemR23 axis enhances ECs proliferation and migration capacity through diversified pathways and mechanisms.

Moreover, CCX832 and the knockdown of chemR23 largely reversed chemerin/chemR23 axis-induced angiogenesis. For example, CCX832 reversed angiogenesis through decreased expression of P38 MAPK, ERK1/2 and matrix metalloproteinases-2/9. Additionally, angiogenesis was also reversed through knockdown of chemR23 by short hairpin RNA (shRNA), along with the down-regulated expression of autophagy-related genes [84–86]. It not only verifies the effect of chemerin on ECs capacity, but also indicates that ChemR23 can be taken as the target to block obesity-related angiogenesis.

#### Elevated expressions and levels of endothelial inflammatory factors

Activated ECs can release a series of inflammatory factors, such as interleukin (IL-6), TNFα and CRP, which leads to abnormal endothelial secretion and inflammation in the blood vessel wall [87]. Besides, elevated expressions of intercellular adhesion molecule 1 and E-selectin have been regarded as the symbols of vascular endothelial activation. It was shown that increased circulating chemerin concentration was accompanied by the elevated level of CRP in obese children and expressions of intercellular adhesion molecule 1 and E-selectin in human coronary artery endothelial cells [88]. Simultaneously, certain inflammatory cytokines such as TNF-α, IL-1β, and IL-6 augmented chemR23 expression in ECs in turn [66]. Moreover, the elevated levels of inflammatory cytokines induced by chemerin/chemR23 axis increased monocyte attachment to ECs [67, 89]. It suggests that the activation of chemerin/chemR23 axis promotes endothelial dysfunction partly through inflammatory mechanisms.

#### Excessive production of reactive oxygen species in ECs

Oxidative stress is associated with multiple pathological processes [90]. As a momentous product of oxidative stress, reactive oxygen species (ROS) is another key factor that contributes to endothelial dysfunction [87, 91]. On one hand, the production of ROS was increased in human aorta endothelial cells with chemerin stimulation, and the chemerin-induced ROS generation was inhibited by N-acetylcysteine, a ROS scavenger [85]. On the other hand, the knock-down of chemR23 decreased the ROS generation [85]. It supported that chemerin/chemR23 axis induced ROS generation. Moreover, as the main source of intracellular ROS production, mitochondria are closely related to chemerin/chemR23 axis [92]. It’s reported that chemerin-treated ECs showed enhanced mitochondrial ROS generation and the mitochondria-targeted antioxidant, Mito-TEMPO significantly suppressed the chemerin-mediated ROS production. Furthermore, oxidative stress induced by chemerin/chemR23 axis subsequently triggers autophagy and apoptosis of ECs, which further impairs the vascular integrity and function [85].

#### Reduced production of nitric oxide in ECs

Nitric oxide (NO) is recognized as an effective vasodilator released by ECs and maintains vascular tone and homeostasis [93]. NO within ECs is mainly produced via endothelial nitric oxide synthase (eNOS) [94]. Chemerin decreased eNOS generation and enhanced NO breakdown, which ultimately led to NO reduction in ECs. Additionally, other potential mechanisms, including eNOS uncoupling, increased O_2_-generation, and reduced NO-dependent cGMP signaling could participate in chemerin/chemR23 axis-related endothelial dysfunction [95].

### Dysfunction of VSMCs

#### Enhanced proliferation and migration capacity of VSMCs

It has been approved that VSMCs proliferation and migration are involved in the pathophysiological process of vascular remodeling [96, 97]. The abnormal vascular structure is accompanied by vascular dysfunction to a great extent. After stimulating VSMCs by chemerin (100 ng/mL) for 20 min, enhanced proliferation and migration capacity of VSMCs could be found. And CCX832 ameliorated VSMCs proliferation and migration. Furthermore, new evidence demonstrated that chemerin/chemR23 axis promoted proliferation and migration of VSMCs through MAPK signaling [98], Akt/ERK signaling [99], endothelin-1 dependent pathway [100], and enhanced autophagy [101]. To some extent, chemerin/chemR23 axis could affect VSMCs capacity through multiple regulatory networks.

#### Excessive apoptosis of VSMCs

Excessive apoptosis is another adverse event for dysfunction of VSMCs in addition to increased proliferation and migration capacity [102]. After stimulating VSMCs by chemerin (100 ng/mL) for 6 h, obvious apoptosis of VSMCs was observed. Similarly, CCX832 improved such apoptosis-associated VSMCs dysfunction [89]. Interestingly, the intervention time of chemerin-induced proliferation and migration was obviously longer than that of chemerin-induced apoptosis. It may indicate that the early effect of chemerin is to promote proliferation and migration of VSMCs, resulting in thickening of the media while the late effect enhanced apoptosis ultimately leading to vascular weakness. This further suggests that chemerin could play different roles in different stages of vascular dysfunction.

#### Augmentation of oxidative stress of VSMCs

Oxidative stress has been identified to exert important effects on deteriorating vascular function through enhanced inflammation, proliferation and migration, apoptosis and so on [103–106]. The generation of ROS was increased in chemerin-incubated VSMCs. However, CCX832 or knockdown of chemR23 by shRNA decreased the production of ROS. It indicates that chemerin/chemR23 axis could induce VSMCs dysfunction partly through an oxidative mechanism [103–106].

#### Up-regulation of Inflammatory factors of VSMCs

There is no doubt that inflammation is involved in the dysfunction of VSMCs and inflammatory factors could impair VSMCs capacity through multiple pathways, such as promoted proliferation and migration, aging and apoptosis [97]. After stimulation with chemerin, the expressions of certain inflammatory cytokines such as IL-1β, IL-6 and monocyte chemoattractant protein-1 (MCP-1) were found to up-regulate in VSMCs. Conversely, CCX832 ameliorated the increase of these inflammatory cytokines [89].

## Cardiovascular diseases mediated by chemerin/chemR23 axis

### Atherosclerosis and acute coronary syndrome (ACS)

The scientists assessed the severity of aortic and coronary arteries by the AHA classification through the angiography completed before the patients died, and then detected chemerin and chemR23 proteins in those arterial specimens by immunohistochemistry. Strong chemerin immunopositivity was observed in PVAT, VSMCs, and foam cells in atherosclerotic lesions, and accompanied by a positive correlation with the severity of atherosclerosis [107]. Nevertheless, chemerin is not recommended as a predictor of human atherosclerosis [108, 109]. Or rather, the predictive value of chemerin immeasurably could hinge on the region of affected arteries and stage of disease [110].

The analogous conclusion in experimental animals was drawn that chemerin accelerated the progression of atherosclerosis in apolipoprotein (Apo) E^−/−^ mice with HFD by adenovirus transfection for knockdown or overexpression of chemerin gene into the aorta or pDCs [111]. In addition, the knockout of chemR23 in pDCs of ApoE^−/−^ mice restricted the formation and progression of atherosclerotic plaque [112]. This pro-atherosclerotic effect is induced by chemerin/chemR23 axis partly through promoting adhesion and migration of ECs [113], inflammation [114], and proliferation of VSMCs.

ACS, including unstable angina pectoris (UAP) and acute myocardial infarction (AMI), is the most feared consequence of partial or complete thrombotic vessel occlusion caused by disruption of a certain coronary atherosclerotic plaque. The plasma chemerin concentration was notably higher in patients with ACS than in those with stable angina pectoris and controls, and the increase of chemerin concentration was synchronized with the elevation of CRP concentration [115–118]. Hence, chemerin could be considered as a novel predictor of ACS. What's more, the average chemerin concentration in AMI is higher than that in UAP [117].

### Hypertension

Compared with healthy controls, adult patients with primary hypertension had significantly higher serum chemerin concentrations [117]. Circulating chemerin concentration also increased even in obese children with elevated systolic blood pressure and not diagnosed with hypertension [119, 120]. Although ample evidence appealed that chemerin concentration was highly positively correlated with blood pressure, more clinical trials are needed to support the view prop up the perspective that chemerin is a predictor of human obesity-induced hypertension.

Obese rats with elevated serum chemerin concentrations showed a tendency of susceptibility to hypertension [48]. There was a certain drop in mean and systolic blood pressures in the chemerin knockout rats [121]. Besides dysfunctions of ECs, VSMCs and PVAT, chemerin/chemR23 axis induced hypertension also through enhanced arterial contraction [26, 38, 47] and sensitivity of the sympathetic nervous system [122].

### Pulmonary arterial hypertension (PAH)

The protein expressions of chemerin and chemR23 were elevated in PAH model rats. And chemerin-9 (chemR23 agonist) induced contraction of the isolated intrapulmonary artery through increasing chemR23 protein expression in VSMCs [123]. Although animal experiments indicated that chemerin/chemR23 axis was involved in the development of PAH, there was still a lack of abundant clinical observations to prove this correlation in patients with PAH.

Aortic valve (AV) stenosis

Degenerative AV stenosis is one of the most common cardiovascular diseases currently in the elderly, which is classified into mild, moderate, severe according to the stricture degree of AV. In comparison with the controls, serum chemerin concentration was observably higher in patients with mild and moderate AV stenosis. Interestingly enough, the patients with the severe stenosis had lower circulating chemerin concentration than those in the mild and moderate stenosis, for which inactivation of inflammation might account [123, 124]. So, chemerin was proposed as a good predictor for mild AV stenosis and chemerin > 38.60 ng/mL was selected as the cut-off point for the diagnosis of mild AV stenosis. [125].

### Abdominal aortic aneurysm (AAA)

AAA is a progressive segmental abdominal aortic dilation. The circulating chemerin concentration was higher in patients suffering from AAA than healthy controls. Besides, compared with the normal abdominal aortic samples, higher mRNA expressions of chemerin and chemR23 were detected in the focus tissues from patients with AAA [123]. This demonstrated that chemerin/chemR23 axis was involved in AAA progression.

**Fig. 2:**
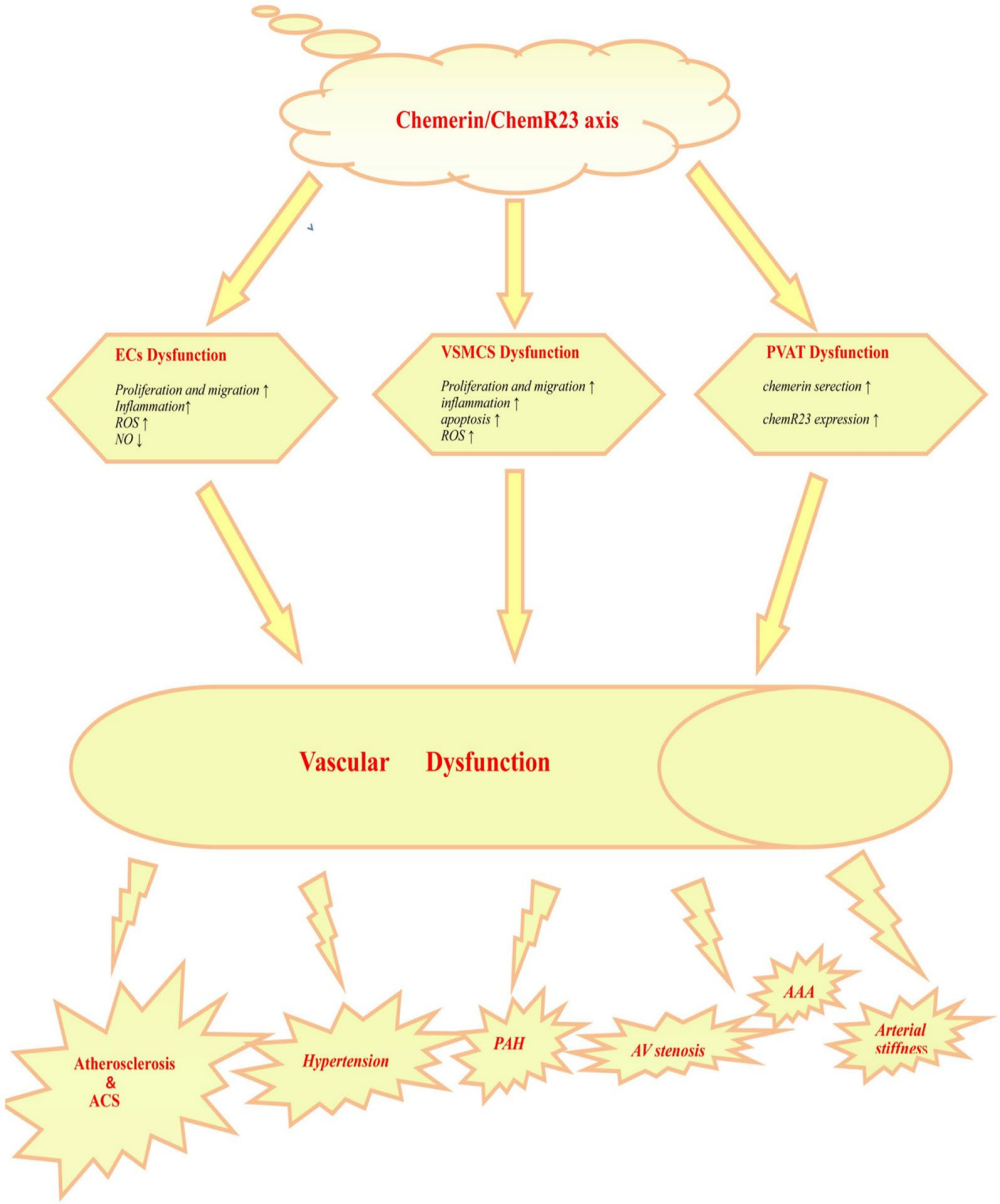
The potential pathogenic effects of chemerin/chemR23 axis on vascular dysfunction and cardiovascular diseases. The schematic figures indicate that chemerin/chemR23 axis leads to vascular dysfunction through diversified pathways, including ECs (enhanced proliferation and migration, increased inflammation, decreased NO production and augmented oxidative stress), VSMCs (enhanced proliferation and migration, increased inflammation, excessive apoptosis, augmented oxidative stress) and PVAT dysfunction (enhanced chemerin secretion and increased chemR23 expression), which further results in various vascular-related diseases

In previous animal experiments, scientists found that DIO mice and ob/ob mice were more prone to AAA than WT mice [126, 127], but the potential mechanisms have not been explored yet. Therefore, we reasonably speculate that chemerin/chemR23 axis could partly promote the formation of AAA in obese mice. (Fig. 2).

### Arterial calcification and arterial stiffness

Arterial calcification and arterial stiffness are independent predictors of cardiovascular risk and mortality. Both processes reinforce one another, creating a vicious cycle. However, chemerin/chemR23 axis seemingly induced contradictory results in the occurrence and progression of arterial calcification and arterial stiffness. On one hand, there was a significantly positive correlation between the circulating chemerin concentration and arterial stiffness in obese patients [120, 128, 129]. On the other hand, chemerin-9 (chemR23 agonist) increased the expression of a calcification inhibitor, matrix gla protein, and reduced phosphate-induced calcification in VSMCs. And the aforementioned effect on arterial calcification mediated by chemerin-9 was not observed VSMCs lacking chemR23. It indicates that chemerin/chemR23 axis may restrain the occurrence and development of arterial calcification [130].

## Future therapeutics for chemrin/chemR23 axis

Although we have a general idea of the significant role of chemerin/chemR23 axis in obesity-related vascular dysfunction, it remains a pity that there is almost no drug circulating in the market targeting chemerin/chemR23 axis. Through a variety of related studies, several drugs targeting chemerin/chemR23 axis are still considered to have potential and feasibility for use in humans. They include CCX832, RvE1, chemerin-9, chemerin ASO, and nano-antioxidant.

### CCX832

Numerous in vivo and in vitro studies have revealed that chemR23 inhibitor, CCX832, greatly reversed chemerin/chemR23 axis-induced vascular dysfunction.

The effect of CCX832 on improving chemerin/chemR23 axis-induced vascular dysfunction has been verified in various cells and in vitro vessels from multiple sources. For example, CCX832 improved chemerin-induced vascular inflammation in human microvascular ECs and human aorta ECs [60, 66]. CCX832 reversed oxidative stress in human aortic smooth muscle cells and VSMCs from mesenteric arteries of C57BL/6 J mice [38, 47]. Besides, CCX832 inhibited abnormal contraction in the isolated rat mesenteric artery, rat thoracic aorta, human pulmonary artery and human coronary artery [38, 131, 132].

Moreover, oral and intravenous administration of CCX832 ameliorated vascular insulin sensitivity in diabetic obese db/db mice and DIO mice [60]. Although CCX achieved phased success in phase 1 clinical trial of patients with psoriasis in 2012, its development was discontinued in 2013 for unknown reasons. Even so, CCX832 was still used in animal and cell experiments and obtained good experimental results. This manifests that CCX832 does play an essential role in blocking chemerin/chemR23 axis signaling and improving the chemerin/chemR23 axis-induced vascular dysfunction. Hence, we should strengthen the development of chemR23 antagonists to find other chemR23 antagonists that can not only replace CCX832 in experiments but also have safety and effectiveness in humans.

### RvE1

RvE1, a new bioactive oxygenated product of EPA, is proved to be another ligand of chemR23. Meanwhile, RvE1 is a specialized pro-resolving mediator (SPMs), which are involved in promoting the resolution of inflammation [133, 134]. RvE1 plays a crucial role in improving vascular function and reducing cardiovascular risk through its downstream specific receptor axis, namely ERV1/chemR23 [135, 136].

A mass of in vivo and in vitro studies have demonstrated that RVE1 exerts powerful cardiovascular benefits, including inhibiting the progression of atherosclerotic plaques [137, 138], reducing the formation of new intima [139], preventing vascular endothelial injury, regulating vasoconstriction [132], restraining vascular calcification [140, 141] and so on. What’s more, multiple clinical trials have also supplemented the cardiovascular benefits of EPA [142–145]. Based on this, RvE1 is speculated rationally to redress vascular dysfunction partly through competing with chemerin for the binding site of chemR23.

### Chemerin-9 and other chemerin isoforms

Chemerin-9, also known as C9, is a synthetic small molecule fragment of chemerin. Although it is biologically active as an analog of chemerin and an agonist of chemR23. Chemerin-9 seemingly plays an anti-inflammatory and positive role in regulating vascular function in most cases, which is the exact opposite of chemerin.

A study described that chemerin-9 attenuated the formation of AAA in obese mice [146]. And the infusion of chemerin-9 significantly decreased the areas of aortic atherosclerotic lesions, accompanied by the reduction of TNF-α [147]. It is well known that chemerin-9 improved vascular function. But interestingly, some studies revealed that chemerin-9 might be involved in the evolvement of hypertension and PAH by inducing arterial contraction [147]. The detailed mechanism is worth exploring.

In addition to chemerin-9, there are other active fragments, such as chemerin-13 (C13). Chemerin-13, similar to chemerin-9, is also an agonist of chemR23, but few studies have been conducted on the treatment of chemerin/chemR23 axis-induced vascular dysfunction with it. More studies are needed to explore the difference between chemerin-13 and chemerin-9 in regulating vascular function.

It will necessitate investigations of the differences between chemerin-13 and chemerin-9 in regulating vascular function and further explorations of the therapeutic possibilities of more fragments or isoforms of chemerin.

### Chemerin antisense oligonucleotide

ASO is a class of molecular drugs that inhibit the expression of target gene DNA or mRNA by sequence-specific binding and regulation at the gene level. It has been found that chemerin ASO with whole-body activity dramatically decreases blood pressure in rats [57, 148]. This opens up the possibility of chemerin ASO in the treatment of chemerin/chemR23 axis-induced vascular dysfunction in humans. Hence, the experimental and clinical studies of chemerin ASO are strongly advocated to provide more diversified options for the treatment of chemerin/chemR23 axis-induced dysfunction.

### Nano-antioxidant

Considering the critical role of oxidative stress on the vascular dysfunction induced by chemerin/chemR23 axis, inhibition of ROS production can reduce chemerin expression to a certain extent, further improving vascular function. Therefore, the application of antioxidants provides a novel direction for the treatment of chemerin/chemR23 axis-induced vascular dysfunction. To date, the effectiveness of traditional antioxidants has been limited for many reasons, such as gastrointestinal degradation, first-pass effect, and/or instability during storage [149]. The combination of nanotechnology and antioxidants can effectively avoid these problems. Nano-antioxidants not only have higher physicochemical stability as well as biological activity, but also have improved ROS scavenging ability [92]. Recently, several types of these nano-antioxidants have demonstrated potential utility in nanomedicine [150, 151]. Thus, The application of nano antioxidant technology is expected to ameliorate obesity-related vascular dysfunction.

## Conclusion

**Fig. 3:**
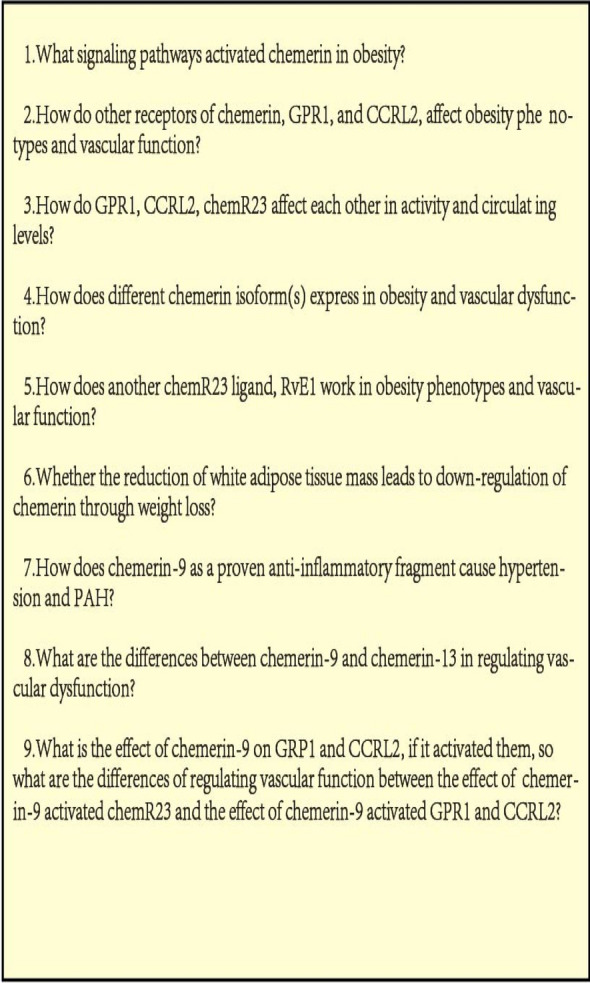
Unsettle experimental questions

The significance of chemerin has been illustrated in many researches. Circulating chemerin concentrations are related to the development and progression of multiple system diseases and even are used as a biomarker for the diagnosis of cardiovascular diseases like ACS, AV stenosis, AAA, and so on. The chemerin/chemR23 axis is a complex network strictly involved in the occurrence and development of obesity and regulation of vascular function, which is supported by abundant rationale from clinical and experimental observations. However, the concrete mechanisms and pathways of chemerin/chemR23 axis displaying in these disorders remain a mystery and worth exploring. Thus, uncovering the answer to key questions (Fig. 3) probably conduces to deeply understand and better define the relationship among this axis, obesity, and vascular dysfunction. Moreover, it is helpful to seek the possibility of other treatments in addition to CCX832, RvE1, chemerin ASO, chemerin-9, nano-antioxidants even other alternative and salutary isoforms of chemerin.

**Table 1:**
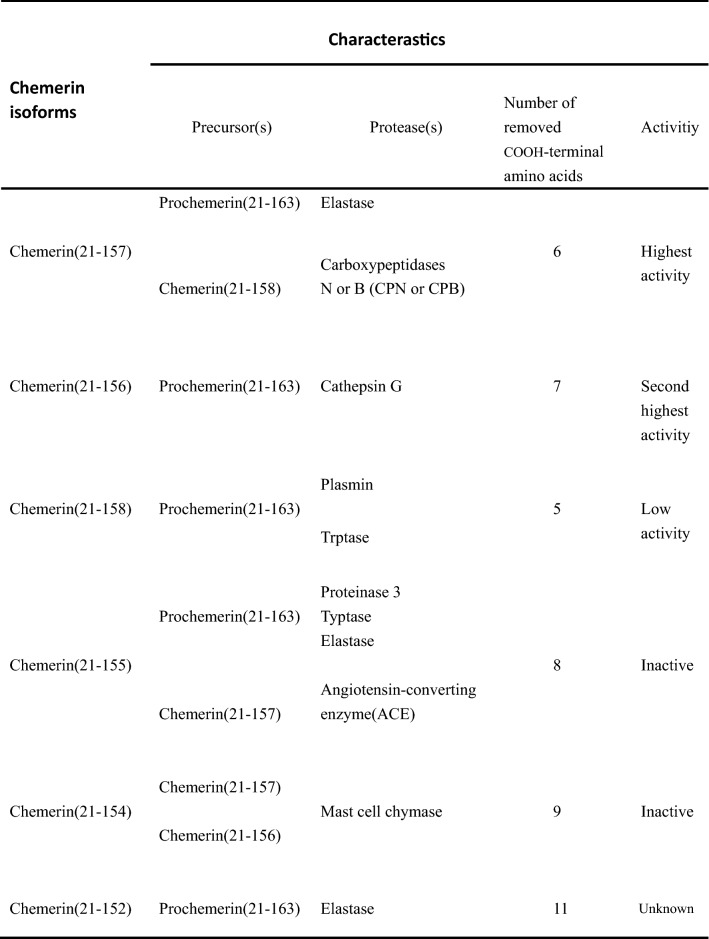
Characteristics of different chemerin isoforms

## Data Availability

Not applicable.

## Abbreviations

WAT: White adipose tissue
PVAT: Perivascular adipose tissue
GTP: Guanosine 5'-triphosphate
GPCR: GTP-binding protein-coupled receptors
CMKLR1: Chemokine like receptor 1
CPN: Carboxypeptidases N
CPB: Carboxypeptidases B
GPR1: G protein-coupled receptor 1
CCRL2: C–C motif receptor-like 2
RvE1: Resolvin E1
EPA: Eicosapentaenoic acid
ω-3 PUFAs: Omega-3 polyunsaturated fatty acids
BRIGHT: British Genetics of Hypertension
CCL: C–C motif chemokine ligand
BMI: Body mass index
SAT: Subcutaneous adipose tissue
VAT: Visceral adipose tissue
WHR: Waist-to-hip ratio
TNFα: Tumor necrosis factor α
CRP: C-reactive protein
HFD: High-fat diet
DIO: Diet-induced-obesity
pDCs: Plasmacytoid dendritic cells
ECs: Endothelial cells
VSMCs: Vascular smooth muscle cells
ASO: Antisense oligonucleotides
ERK: Extracellular regulated protein kinases
MAPK: Mitogen-activated protein kinase
shRNA: Short hairpin RNA
IL: Interleukin
ROS: Reactive oxygen species
NO: Nitric oxide
eNOS: Endothelial nitric oxide synthase
Apo: Apolipoprotein
MCP-1: Monocyte chemoattractant protein-1
UAP: Unstable angina pectoris
AMI: Acute myocardial
PAH: Pulmonary hypertension
AV: Aortic valve
AAA: Abdominal aotic aneurysm
